# Three-dimensional assessment of the anterior and inferior loop of the inferior alveolar nerve using computed tomography images in patients with and without mandibular asymmetry

**DOI:** 10.1186/s12903-021-01424-3

**Published:** 2021-02-16

**Authors:** Jae-Young Kim, Michael D. Han, Kug Jin Jeon, Jong-Ki Huh, Kwang-Ho Park

**Affiliations:** 1 Department of Oral and Maxillofacial Surgery, Gangnam Severance Hospital, Yonsei University College of Dentistry, 211 Eonju-ro, Gangnam-gu, Seoul, 06273 Korea; 2 Department of Oral and Maxillofacial Surgery, University of Illinois at Chicago College of Dentistry, Chicago, IL USA; 3 Department of Oral and Maxillofacial Radiology, Yonsei University College of Dentistry, Seoul, Korea

**Keywords:** Facial asymmetry, Inferior alveolar nerve, Three-dimensional analysis, Computed tomography

## Abstract

**Background:**

The purpose of this study was to investigate the differences in configuration and dimensions of the anterior loop of the inferior alveolar nerve (ALIAN) in patients with and without mandibular asymmetry.

**Method:**

Preoperative computed tomography images of patients who had undergone orthognathic surgery from January 2016 to December 2018 at a single institution were analyzed. Subjects were classified into two groups as “Asymmetry group” and “Symmetry group”. The distance from the most anterior and most inferior points of the ALIAN (IANant and IANinf) to the vertical and horizontal reference planes were measured (dAnt and dInf). The distance from IANant and IANinf to the mental foramen were also calculated (dAnt_MF and dInf_MF). The length of the mandibular body and symphysis area were measured. All measurements were analyzed using 3D analysis software.

**Results:**

There were 57 total eligible subjects. In the Asymmetry group, dAnt and dAnt_MF on the non-deviated side were significantly longer than the deviated side (*p* < 0.001). dInf_MF on the non-deviated side was also significantly longer than the deviated side (*p* = 0.001). Mandibular body length was significantly longer on the non-deviated side (*p* < 0.001). There was no significant difference in length in the symphysis area (*p* = 0.623). In the Symmetry group, there was no difference between the left and right sides for all variables.

**Conclusion:**

In asymmetric patients, there is a difference tendency in the ALIAN between the deviated and non-deviated sides. In patients with mandibular asymmetry, this should be considered during surgery in the anterior mandible.

## Background

The inferior alveolar nerve (IAN) travels through the mandibular canal and innervates the lower lip, chin, mandibular teeth and the anterior gingiva and alveolar mucosa. The last part of the IAN runs anterior and inferior to the mental foramen and forms an anterior loop. It has been reported that an anterior loop of the inferior alveolar nerve (ALIAN) can be observed in about 40–94% of cases [1–4].

For this reason, iatrogenic damage of the IAN can occur during procedures such as genioplasty, implant surgery near the mental foramen, IAN lateralization, and open reduction and internal fixation of fractures. According to previous studies, IAN damage has been reported to be 17–38% after genioplasty [5–7]. Hwang et al. recommended the level of a genioplasty osteotomy be at least 4.5 mm below the mental foramen to avoid iatrogenic damage [8]. For dental implant surgery, a 4–6 mm safety margin from the anterior border of mental foramen has been recommended [2, 9]. However, Filo et al. reported that it is difficult to provide a guideline for the safety margin due to varying lengths of the anterior loop [10].

Several authors have reported differences in the inferior alveolar nerve in facial asymmetry on the deviated and non-deviated sides [11, 12]. Yoon et al. reported that both the anterior and inferior of the ALIAN are longer in mandibular prognathism than in mandibular retrognathism [13].They also concluded that the length of the anterior loop can increase as the mandibular subunit becomes longer [13]. In this regard, variations may exist in the ALIAN in asymmetric mandibles, which may warrant different safety margins bilaterally.

The purpose of this study was to investigate the differences in configuration and dimensions of the anterior loop of the inferior alveolar nerve (ALIAN) in patients with and without mandibular asymmetry.

## Methods

### Subjects

Records of patients who underwent a computed tomography (CT) scan and orthognathic surgery from January 2016 to December 2018 in the Department of Oral and Maxillofacial Surgery at Gangnam Severance Hospital were reviewed in this retrospective study. Patients with missing maxillary first molar or mandibular central or lateral incisor, cleft lip and palate, history of mandibular trauma affecting the body or ramus length, or lateral chin deviation of 2.5–3.5 mm on CT analysis were excluded. CT images were obtained with a CT scanner (Siemens definition AS+, Siemens, Germany) by following parameters: 1-mm slice thickness, 7-s scan time, 120 kV, and 90 mAs. Those whose CT scans lacked clearly identifiable mandibular canals were also excluded.

This study was approved by the institutional review board (IRB) of Gangnam Severance Hospital (approval No. #3-2019-0236) and complied with the tenets of the Declaration of Helsinki. Written or verbal informed consent was waived by the IRB of Gangnam Severance Hospital, as this study had a retrospective design and all data were analyzed anonymously.

### Reference points and planes

Reference points and planes were summarized in Table 1. All landmarks and reference planes were identified using multiplanar reconstructed CT image using image analysis software (Mimics research 21.0, Materialise, Belgium).

**Table 1:** Reference points and planes

Landmarks (abbreviation)	Description
Points	
Orbitale (Or)	The lowest point on the lower margin of the bony orbit (OrR, right side; OrL, left side)
Porion (Po)	The highest point of the skeletal external auditory meatus (PoR, right side; PoL, left side)
Basion (Ba)	Midpoint of the anterior margin of the foramen magnum on the occipital bone
Crista galli (Cg)	The most superior point of crista galli located in the ethmoid bone
Mesiobuccal cusp (MBC)	Mesiobuccal cusp of maxillary first molar (MBCR: right; MBCL; left)
Mandibular foramen (ManF)	The center point of the opening of the inferior alveolar nerve canal
Mental foramen (MF)	The center of mental foramen (MFR, right side; MFL, left side)
Lower incisor (L1)	The midpoint of the incisal edge of the mandibular central incisors
IANant	The most anterior point of the anterior loop of the inferior alveolar nerve
IANinf	The most inferior point of the anterior loop of the inferior alveolar nerve
Planes	
Mid-axial plane (MAP)	A plane passing through OrR, OrL and PoL
Mid-sagittal plane (MSP)	A plane passing through Cg, Ba and normal to MAP
Coronal plane (COP)	A plane passing through Ba, and normal to MAP and MSP
Occlusal plane (OP)	A plane passing through the bilateral mesiobuccal cusp of maxillary first molar and the L1
Mandibular sagittal plane (MnSP)	A plane passing through ManF, MF and normal to OP
Horizontal reference plane (HRP)	A plane passing through MF and parallel to OP
Vertical reference plane (VRP)	A plane passing through MF and normal to OP and MnSP

#### Reference points

Porion (Po), orbitale (Or), pogonion (Pog), mental foramen (MF), mandibular foramen (ManF), mesiobuccal cusp of maxillary first molar (MBC), midpoint of the incisal edge of the mandibular central incisors (L1), basion (Ba), and crista galli (Cg) were set as the reference points. The most anterior and the most inferior points of ALIAN were set as IANant and IANinf, respectively.

#### Reference planes

**Fig. 1:**
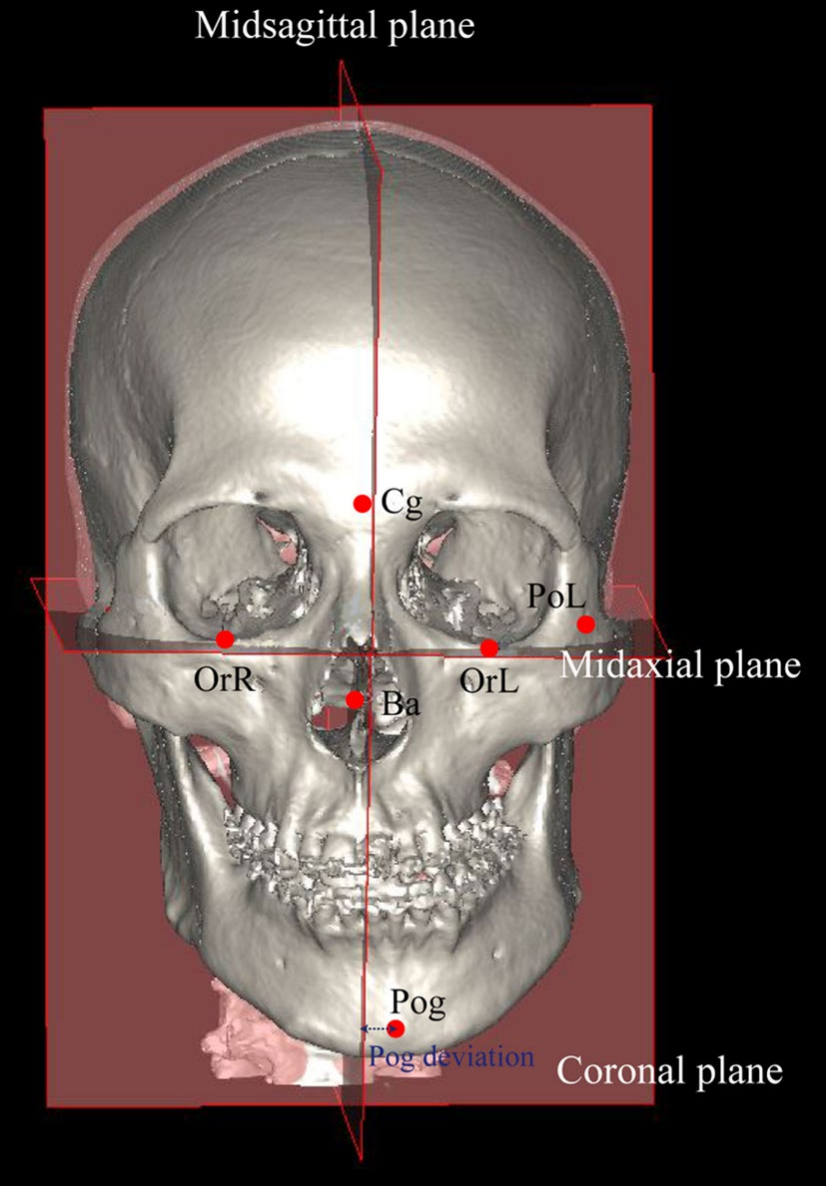
Reference points and plane for diagnosis of facial asymmetry. Facial asymmetry was defined when distance from Pog to midsagittal plane (Pog deviation) was over 3.5 mm. OrR, Orbitale right; OrL, Orbitale left; PoL, Porion left; Cg, Crista galli; Ba, Basion, Pog, Pogonion

The mid-axial plane, mid-sagittal plane, and coronal plane were also determined. “Midaxial plane” was defined as the plane passing through bilateral Or (OrR, OrL) and left Po (PoL). “Midsagittal plane” was defined as the plane perpendicular to the midaxial plane with passing through Ba and Cg. A plane perpendicular to the midaxial plane and midsagittal plane and bypassing Ba was defined as “Coronal plane” (Fig. 1) Occlusal plane was defined as the plane passing through the bilateral mesiobuccal cusp of maxillary first molar and the L1.

**Fig. 2:**
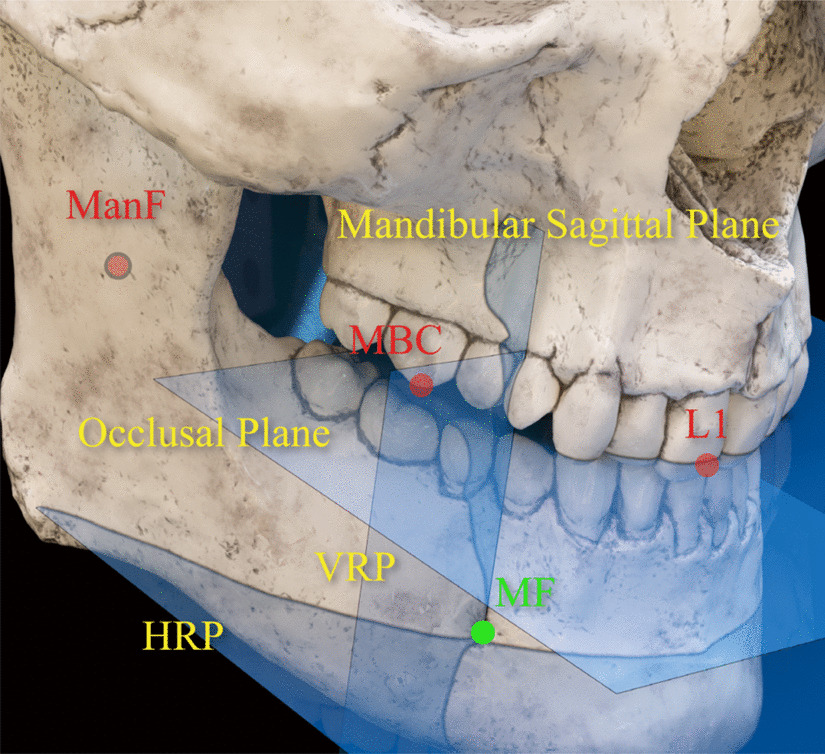
Reference planes for distance measurement of the anterior loop of inferior alveolar nerve (ALIAN) (illustrated image). Occlusal plane was defined as a plane passing through L1 and the bilateral mesiobuccal cusp of the maxillary first molar. A plane passing through ManF, MF and perpendicular to occlusal plane was defined as the mandibular sagittal plane. The vertical reference plane (VRP) was defined as a plane passing through MF and perpendicular to occlusal plane and MnSP. The horizontal reference plane (HRP) was defined as a plane passing through MF and parallel to occlusal plane. MBC, Mesiobuccal cusp of maxillary first molar; ManF, Center of the opening of the inferior alveolar nerve canal; MF, Center of the mental foramen; HRP, Horizontal reference plane; VRP, Vertical reference plane

To evaluate the anterior and inferior distance of the anterior loop of IAN, vertical and horizontal reference planes were set on mandible. First, a plane passing through ManF, MF and perpendicular to occlusal plane was defined as the mandibular sagittal plane. The vertical reference plane (VRP) was defined as a plane passing through MF and perpendicular to occlusal plane and mandibular sagittal plane. The horizontal reference plane (HRP) was defined as a plane passing through MF and parallel to occlusal plane (Fig. 2).

Measurements using a three-dimensional analysis program were performed twice over at least 1 week by an experienced oral and maxillofacial surgeon. This examiner was blinded to the subjects’ groups.

### Definition of mandibular asymmetry

The distance from Pog to the midsagittal plane (Pog deviation) was measured. (Fig. 1) All patients were divided into two groups depending on Pog deviation as “Asymmetry” and “Symmetry” groups. Asymmetry was classified based on 3.0 mm of Pog deviation [14]. To increase the reliability of the study results, Pog deviation between 2.5 and 3.5 mm were excluded. Finally, patients were classified as “Asymmetry” group (Asy) when Pog deviation was greater than 3.5 mm. “Symmetry” group (Sym) was defined when Pog deviation was less than 2.5 mm.

### Analysis of the anterior loop of IAN

Four outcome variables were defined for analysis of the ALIAN.

**Fig. 3:**
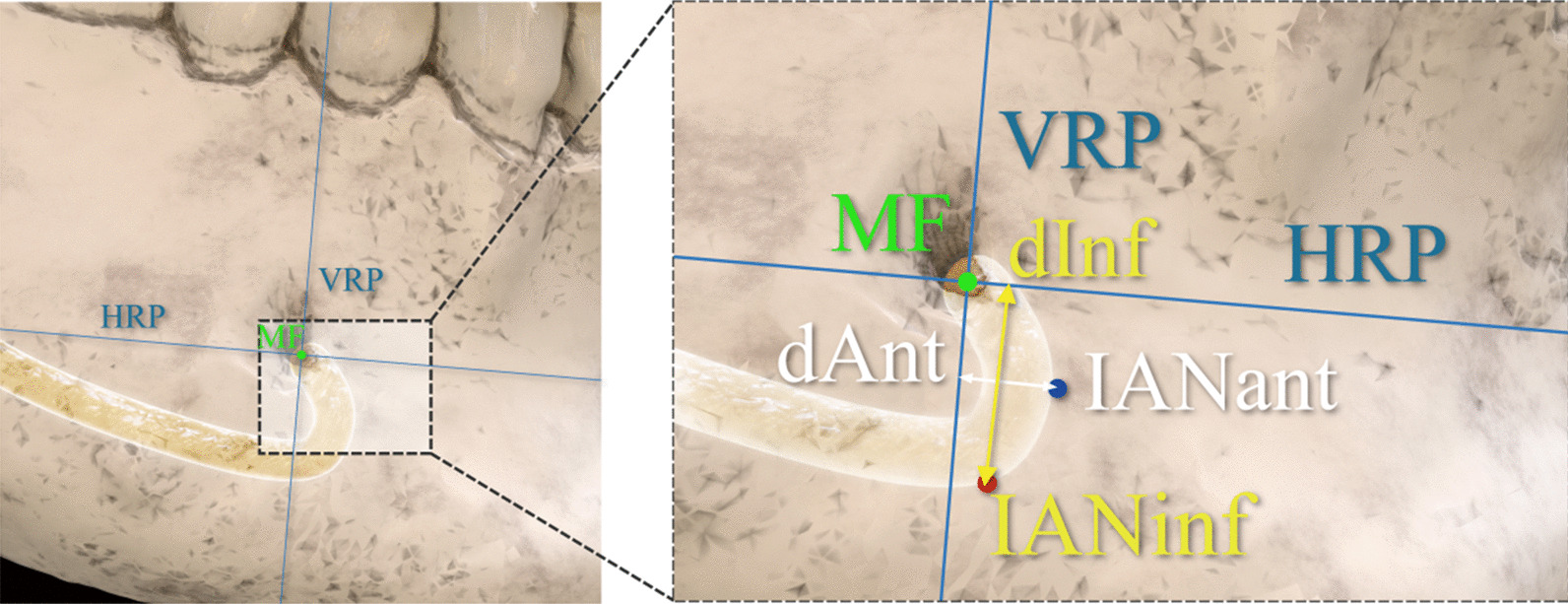
Measurement of dAnt and dInf (illustrated image). dAnt refers to the distance from the most anterior point of the ALIAN (IANant) to the vertical reference plane (VRP). dInf refers to the distance from the most inferior point of ALIAN (IANinf) to the horizontal reference plane (HRP)

1. dAnt: The distance from IANant to VRP (Fig. 3)
2. dInf: The distance from IANinf to HRP (Fig. 3)
3. dAnt_MF: The distance from IANant to MF
4. dInf_MF: The distance from IANinf to MF

dAnt_MF and dInf_MF, were calculated using the Elucidean formula. For example, dAnt_MF were calculated as follows;

1. The distance from IANant and MF to midaxial plane, coronal plane, and midsagittal plane were measured. (dMAP_Ant, dMAP_MF, dCOP_Ant, dCOP_MF, dMSP_Ant, dMSP_MF).
2. Then, the difference was calculated in each plane. (e.g.dMAP_Ant– dMAP_MF).
3. The sum of the squares of each length was calculated, and the value of the square root of the positive is obtained as shown in the following equation.
  $$dAnt_MF = \sqrt{{(dMAP\_Ant-dMAP\_MF)}^{2}+{(dCOP\_Ant-dCOP\_MF)}^{2}+{(dMSP\_Ant-dMSP\_MF)}^{2}}$$
  dInf_MF was also calculated in the same way. Each value was measured and calculated on both right and left side. In the Asy group, they were re-classified as the deviated and non-deviated side.

### Measurements of mandibular body and symphysis length [15]

Two outcome variables were defined for analysis the length of mandibular body and symphysis. They were measured automatically by image analysis software.

1. Mandibular body length: The distance from ManF to MF, which indicates the length of mandibular body
2. Symphysis length: The distance from MF to Pog, which indicates the length of symphysis

Each value was measured on both the right and left side. In the Asy group, they were re-classified as the deviated and non-deviated side.

### Statistical analysis

Age and sex were analyzed with Mann–Whitney test and chi-square test between the two groups. Intraclass correlation coefficient between two measurements was evaluated. Six outcome variables (dAnt, dInf, dAnt_MF, dInf_MF, mandibular body length and symphysis length) were analyzed by paired t-test in both groups. The analysis was performed using the average values of two measurements. The relationships between each distance measurements (dAnt, dInf, dAnt_MF, dInf_MF) and mandibular unit (mandibular body length and symphysis length) were analyzed by Pearson’s correlation analysis. The relationships between the difference between the non-deviated side and deviated side (i.e. non-deviated side–deviated side or left–right side) and degree of asymmetry in each variables were also analyzed by Pearson’s correlation analysis. If the normality test was not satisfied, the analysis was performed using the nonparametric test methods such as Wilcoxon signed-rank test and Spearman's rank correlation coefficient analysis. In the Asy group, the deviated and non-deviated sides were compared. In the Sym group, the right and left sides were compared. Statistical analysis was performed using SPSS version 23.0 (IBM Corp, Armonk, NY). A *p*-value of less than 0.05 was considered statistically significant.

## Results

Eighty-one subjects were initially identified, and 24 were excluded as they did not meet the inclusion criteria. Of the 57 subjects meeting the inclusion criteria, 33 were classified as “Asy group” and 24 were classified as “Sym group”. There were 14 male and 19 female patients (mean age: 22.64 ± 5.33 years old) in Asy group. In Sym group, 15 male and 9 female patients (mean age: 22.75 ± 3.97 years old). There was no significant difference in age (*p* = 0.333) and sex (*p* = 0.134) between the two groups. (Table 2) The intraclass correlation coefficient ranged from 0.705 to 0.976, which was evaluated as "excellent" in most variables and "good" in some variables.

**Table 2:** Clinical characteristics of the patients

	Asy group (n = 33)	Sym group (n = 24)	*p*-value
Age^†^	22.64 ± 5.33	22.75 ± 3.97	0.333
Sex (n, %)			0.134
Male	14 (42.4)	15 (62.5)	
Female	19 (57.6)	9 (37.5)	

^†^Mann–Whitney test

### Comparisons of distance of ALIAN (dAnt, dAnt_MF, dInf, and dInf_MF), and mandibular unit (mandibular body length and symphysis length) on deviated and non-deviated side (left and right side) in both groups (Table 3)

**Table 3:** Comparison of length of ALIAN, length of mandibular subunits on Asymmetry and Symmetry groups

		Asy group (n = 33)			Sym group (n = 24)	
		Mean ± SD (Min, Max)	*p*-value		Mean ± SD (Min, Max)	*p*-value
dAnt	Dev†	1.79 ± 1.04 (0.57, 5.31)	< 0.001*	Left	1.97 ± 1.00 (0.12, 4.98)	0.746
	Ndev†	3.05 ± 1.04 (1.04, 5.43)		Right	1.94 ± 0.89 (0.54, 4.56)	
dAnt_MF	Dev	4.24 ± 1.42 (2.18, 8.35)	< 0.001*	Left†	4.30 ± 1.20 (2.26, 8.01)	0.324
	Ndev	5.32 ± 1.60 (2.69, 9.50)		Right†	4.27 ± 1.28 (1.88, 7.75)	
dInf	Dev	5.09 ± 1.48 (2.46, 8.08)	0.060	Left	5.44 ± 1.12 (3.06, 8.07)	0.501
	Ndev	5.42 ± 1.69 (2.54, 8.72)		Right	5.29 ± 1.14 (3.42, 8.01)	
dInf_MF	Dev	7.06 ± 1.70 (4.37, 10.96)	0.001*	Left	7.27 ± 1.21 (5.46, 9.71)	0.392
	Ndev	7.95 ± 2.03 (5.19, 12.55)		Right	7.12 ± 1.03 (4.94, 9.30)	
Mandibular body length	Dev	62.47 ± 4.51 (55.38, 73.16)	< 0.001*	Left	66.24 ± 5.45 (55.21, 75.51)	0.368
	NDev	65.61 ± 5.25 (55.44, 77.64)		Right	66.74 ± 5.53 (55.25, 76.99)	
Symphysis length	Dev	30.96 ± 2.34 (25.82, 34.88)	0.623	Left	30.63 ± 2.30 (26.63, 35.03)	0.158
	NDev	30.82 ± 2.22 (26.67, 35.00)		Right	31.17 ± 2.26 (26.63, 35.50)	

ALIAN, anterior loop of inferior alveolar nerve; Asy, Asymmetry; Sym, Symmetry; dAnt, distance from IANant to VRP; dAnt_MF, distance from IANant to MF; dInf, distance from IANinf to HRP; dInf_MF, distance from IANinf to MF; Asy, Asymmetry; Sym, Symmetry; Dev, deviated side; Ndev, non-deviated side

^†^Wilcoxon signed-rank test

^*^Indicates *p* < 0.05

In the Asy group, the distance between IANant and VRP (dAnt) on the deviated (3.05 ± 1.04 mm) and the non-deviated (1.79 ± 1.04 mm) side showed a statistically significant difference (*p* < 0.001). dAnt_MF on the deviated side was 4.24 ± 1.53 mm, and on the non-deviated side was 5.32 ± 1.60 mm, which also showed a statistically significant difference (*p* < 0.001). dInf was longer on the non-deviated side than on the deviated side, but there was no statistically significant difference between the deviated and non-deviated sides (*p* = 0.060). In the Sym group, there were no statistically significant differences in the lengths of the left and right sides.

The mandibular body length showed a significant difference between the deviated side (62.47 ± 4.51 mm) and the non-deviated side (65.61 ± 5.25 mm) in the Asy group (*p* < 0.001). However, the symphysis area did not show any difference (*p* = 0.623). In the Sym group, mandibular body length and symphysis length also showed no significant differences between the right and left sides (*p* = 0.420 and *p* = 0.216, respectively).

### Relationship between distance of ALIAN and degree of asymmetry

**Fig. 4:**
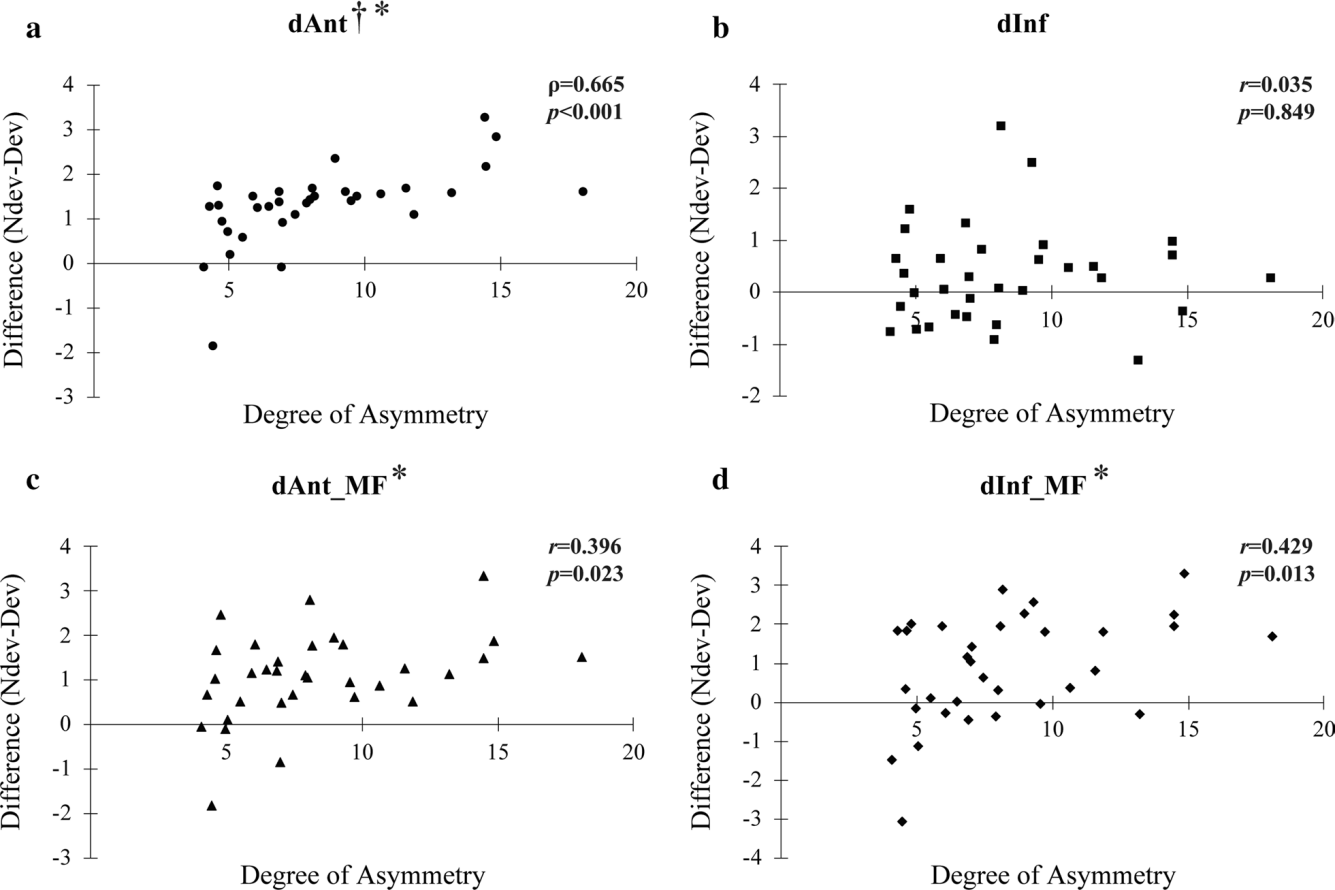
Relationship between degree of asymmetry and difference of non-deviated side and deviated side in Asy group. Ndev, non-deviated side; Dev, deviated side, dAnt, the distance from the most anterior point of the ALIAN (IANant) to vertical reference plane; dInf, the distance from the most inferior point of ALIAN (IANinf) to horizontal reference plane; dAnt_MF, The distance from IANant to the mental foramen; dInf_MF, The distance from IANinf to the mental foramen; *r*, Pearson’s correlation coefficiency; ρ, Spearman’s rho. *p < 0.05. ^†^Spearman’s rank correlation coefficient analysis

**Fig. 5:**
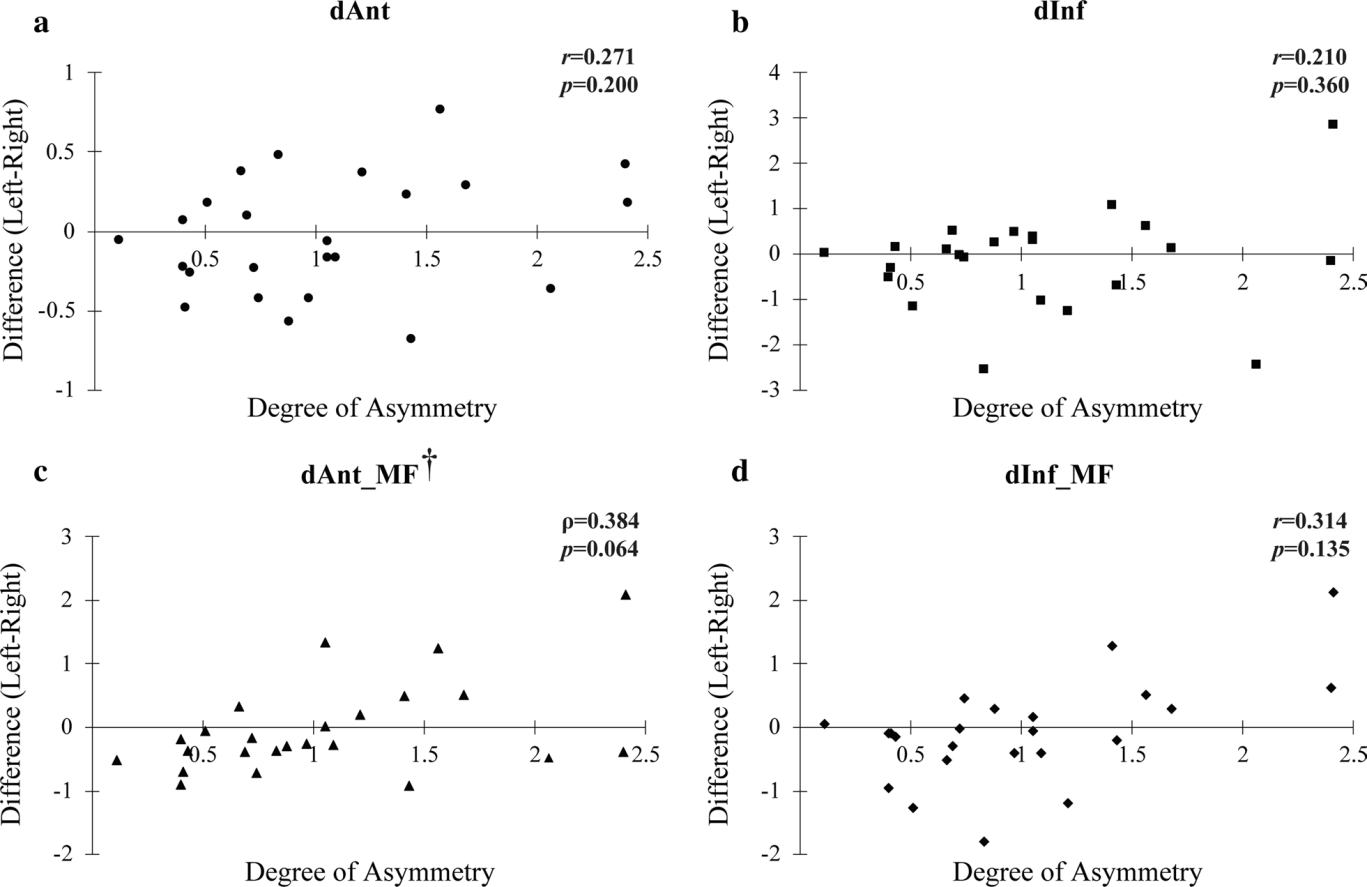
Relationship between degree of asymmetry and difference of left and right side in Sym group. dAnt, the distance from the most anterior point of the ALIAN (IANant) to vertical reference plane; dInf, the distance from the most inferior point of ALIAN (IANinf) to horizontal reference plane; dAnt_MF, The distance from IANant to the mental foramen; dInf_MF, The distance from IANinf to the mental foramen; *r*, Pearson’s correlation coefficiency; ρ, Spearman’s rho. ^†^Spearman’s rank correlation coefficient analysis

In the Asy group, difference between non-deviated and deviated side (i.e. non-deviated -deviated side) had positive correlations with degree of asymmetry in dAnt (ρ = 0.665, *p* < 0.001), dAnt_MF (*r* = 0.396, *p* = 0.023), dInf_MF (*r* = 0.429, *p* = 0.013) with significant difference (Fig. 4). In the Sym group, there was no significant correlation between degree of asymmetry and distance of ALIAN (Fig. 5).

### Relationship between distance of ALIAN and mandibular unit in both groups (Table 4)

**Table 4:** Correlation coefficient analysis between ALIAN and mandibular subunits

	Asy group					Sym group			
	Mandibular body length		Symphysis length			Mandibular body length		Symphysis length	
	Co	*p*	Co	*p*		Co	*p*	Co	*p*
dAnt	0.348*	0.004	0.242	0.050	dAnt	0.321*	0.026	0.177	0.229
dAnt_MF†	0.387*	< 0.001	0.319*	0.009	dAnt_MF	0.139	0.346	0.089	0.549
dInf	0.337*	0.006	0.182	0.144	dInf	− 0.079	0.594	0.062	0.677
dInf_MF	0.413*	0.001	0.261*	0.035	dInf_MF	− 0.041	0.780	0.072	0.626

ALIAN, anterior loop of inferior alveolar nerve; Asy, Asymmetry; Sym, Symmetry; dAnt, distance from IANant to VRP; dAnt_MF, distance from IANant to MF; dInf, distance from IANinf to HRP; dInf_MF, distance from IANinf to MF

^*^Indicates *p* < 0.05

^†^Spearman’s rank correlation coefficient analysis

In the Asy group, mandibular body length had a positive correlation with dAnt, dAnt_MF, dInf, and dInf_MF, with statistical significance. dAnt_MF and dInf_MF also showed a positive correlation with symphysis length with statistical significance. In the Sym group, there were no significant correlations in all parameters except dAnt and mandibular body length.

## Discussion

The purpose of this study was to investigate (1) the differences in configuration and dimensions of the anterior loop of the inferior alveolar nerve (ALIAN) in patients with and without mandibular asymmetry and (2) the relationship with the mandibular subunit. Although the length of the ALIAN on the left and right sides can be compared easily on panoramic radiographs, CT images yield more reliable and accurate measurements, and also show a higher prevalence of the anterior loop than in panoramic radiographs [16, 17]. Thus, in this study, we analyzed CTs taken before orthognathic surgery.

Six variables were evaluated: (1) the distance from IANant to the vertical reference plane (VRP), (2) the distance from IANinf to horizontal reference plane (HRP), (3) the distance from IANant and to MF, (4) the distance from IANinf to MF, (5) the distance from ManF to MF (length of mandibular body), and (6) the distance from MF to Pog (length of symphysis). All items were measured on the left and right sides. The Asy group was reclassified as the deviated side and the non-deviated side to evaluate according to the asymmetry. To minimize errors, measurements were taken twice by one observer. The intraclass correlation coefficient for the repeated measurements ranged from 0.705 to 0.976, which was evaluated as "excellent" in most variables and "good" in some variables [18]. Thus, the data was considered to be reliable.

According to the results of this study, the values in dAnt and dAnt_MF between the deviated and the non-deviated sides showed significant differences in Asy group (*p* < 0.001). There was a significant difference on the deviated and non-deviated sides in dInf_MF in Asy group (*p* = 0.001). This overall shows that the ALIAN deviates less from the mental foramen on the deviated side in asymmetric mandibles. In the Sym group, however, both dAnt and dAnt_MF were not different between the left and right sides.

To analyze whether the differences in the ALIAN between the deviated and non-deviated sides in the Asy group correlated with different mandibular subunits, we measured the length of mandibular body and symphysis based on previous studies [15]. In the Asy group, the length of the mandibular body showed significant differences (*p* < 0.001), whereas the length of symphysis area did not (*p* = 0.623). In the Sym group, both mandibular body length and symphysis length on the left and right sides did not show any difference statistically. These results are consistent with those of You et al. and Park et al., who reported that in facial asymmetry there was no difference in the length of the symphysis but the length of mandibular body [15, 19, 20].

In correlation analysis, dAnt, dInf, dAnt_MF, and dInf_MF showed a positive relationship with mandibular body length with statistical significance in the Asy group. In the Sym group, however, there was no significant positive correlation in all variables except dAnt and mandibular body length as shown in Table 4. Therefore, it seems that the anterior and inferior distances of ALIAN are related to the growth of mandible, especially the growth of mandibular body.

Knowledge of the configuration and dimensions of the ALIAN is important in surgical procedures such as dental implant placement and bone harvesting in the anterior mandible, and genioplasty [10, 21]. The results of this study show differences in the configuration and dimensions of the ALIAN in symmetrical and asymmetrical mandibles, highlighting the need for potentially using different safety margins for certain osteotomy or ostectomy procedures for each side in asymmetric mandibles. Therefore, recognition of the difference of the ALIAN in asymmetric patients is important, especially in a bilateral procedure. While clinicians assess individual anatomy of the ALIAN when three-dimensional imaging is available, knowledge of average configurations and mean dimensions serves as a valuable reference when fine cortications of the mandibular canal are not readily visible, or when three-dimensional imaging is not available. Data from this study could also be of particular use in nerve lateralization for procedures such as inferior border ostectomy in hemimandibular hyperplasia, which require greater three-dimensional knowledge of the anatomy of the ALIAN.

This study has some limitations in that all measurements were made by one observer. Although intra-observer reliability was assessed and found to be satisfactory, inter-observer reliability was not accounted for. In addition, while reference planes are critical to standardize the imaging analysis and measurements, they are somewhat difficult to reproduce completely in actual clinical situations. However, this limitation is not unique to this study, and applies to any clinical situation in which imaging or reference ranges are relied on to determine anatomy without use of adjuncts such as surgical guides or navigation.

## Conclusion

Within the limitation of this study, there is a tendency of difference in ALIAN between the deviated and non-deviated sides in asymmetric patients. Therefore, this should be considered during surgery in the anterior mandible in patients with mandibular asymmetry.

## Data Availability

All data generated or analysed during this study are included in this published article.

## Abbreviations

ALIAN: Anterior loop of the inferior alveolar nerve
MF: Mental foramen
Po: Porion
Or: Orbitale
Pog: Pogonion
ManF: Mandibular foramen
MBC: Mesiobuccal cusp of maxillary first molar
L1: Midpoint of the incisal edge of the mandibular central incisors
Ba: Basion
Cg: Crista galli
IANant: The most anterior point of the ALIAN
IANinf: The most inferior point of the ALIAN
dAnt: The distance from IANant to vertical reference plane
dInf: The distance from IANinf to horizontal reference plane
dAnt_MF: The distance from IANant to the mental foramen
dInf_MF: The distance from IANinf to the mental foramen
